# Supplementary material for: Bioproduction Optimization, Characterization, and Bioactivity of Extracellular Pigment Produced by Streptomyces parvulus
Source: Int J Mol Sci. 2025 Nov 5;26(21):10762. doi: 10.3390/ijms262110762 (PMC12609878; doi:10.3390/ijms262110762)
Supplement: Supplementary file 1 [file ijms-26-10762-s001.zip › ijms-3936377-supplementary.pdf]

Supplementary Material

Article

Bioproduction Optimization, Characterization, and Bioactivity of the Extracellular Pigment Produced by *Streptomyces parvulus*

Laura Daniela Silva-Arias[1,2], Luis Díaz[2,3,*], Ericsson Coy-Barrera[4]

[1] Master in Process Design and Management, School of Engineering, Universidad de La Sabana, Chia 140013, Colombia. laurasiar@unisabana.edu.co (L.D.S.-A.)
[2] Bioprospecting Research Group, School of Engineering, Universidad de La Sabana, Chia 140013, Colombia.
[3] Agroindustrial Production Research Group, Doctorate of Biosciences, School of Engineering, Universidad de La Sabana, Chia 140013, Colombia.
[4] Bioorganic Chemistry Laboratory, Universidad Militar Nueva Granada, Cajicá 250247, Colombia. ericsson.coy@unimilitar.edu.co (E.C.-B.)
* Correspondence: luis.diaz1@unisabana.edu.co (L.D.)

Content

		page
**Figure S1.**	UV-Vis spectra of the S145-derived pigment-rich fraction	2
**Figure S2.**	FTIR spectra of the S145-derived pigment-rich fraction	2
**Figure S3.**	LC-MS-derived total ion chromatogram (negative mode) of the pigment-rich fraction	3
**Figure S4.**	High-resolution mass spectra (HRMS) of the main features for the total ion chromatogram of the S145-derived pigment-rich fraction.	3

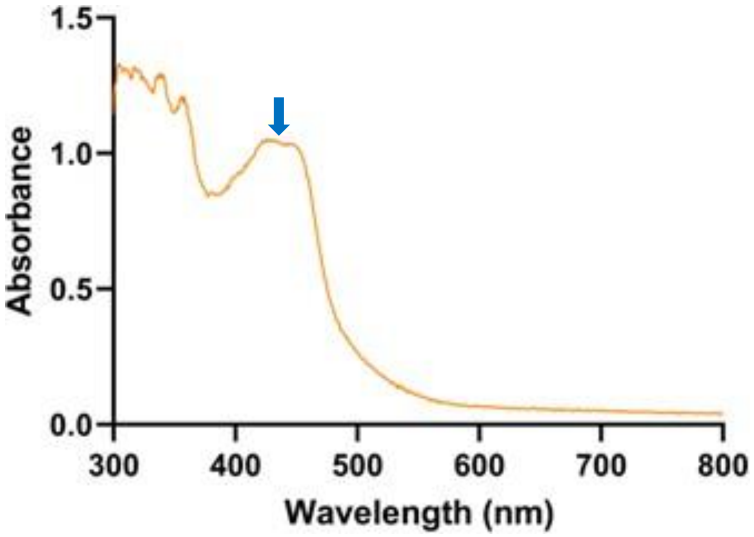

Figure S1. UV-Vis spectra of the S145-derived pigment-rich fraction. The blue arrow (450 nm) shows the wavelength of maximum absorption for the pigment.

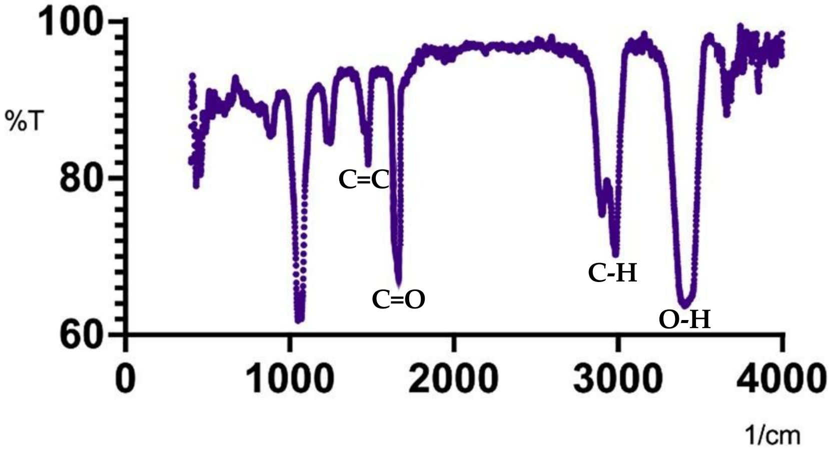

Figure S2. FTIR spectra of the S145-derived pigment-rich fraction

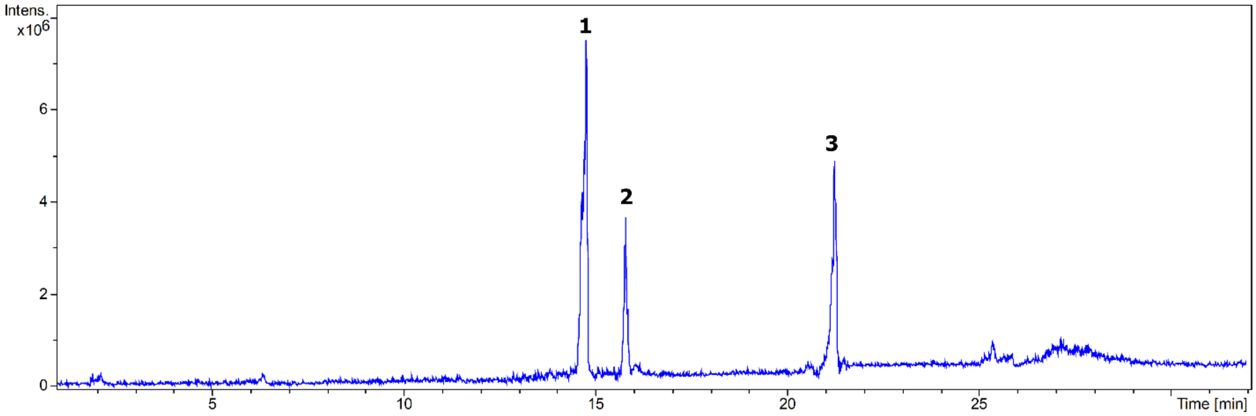

Figure S3. LC-MS-derived total ion chromatogram (negative mode) of the pigment-rich fraction.

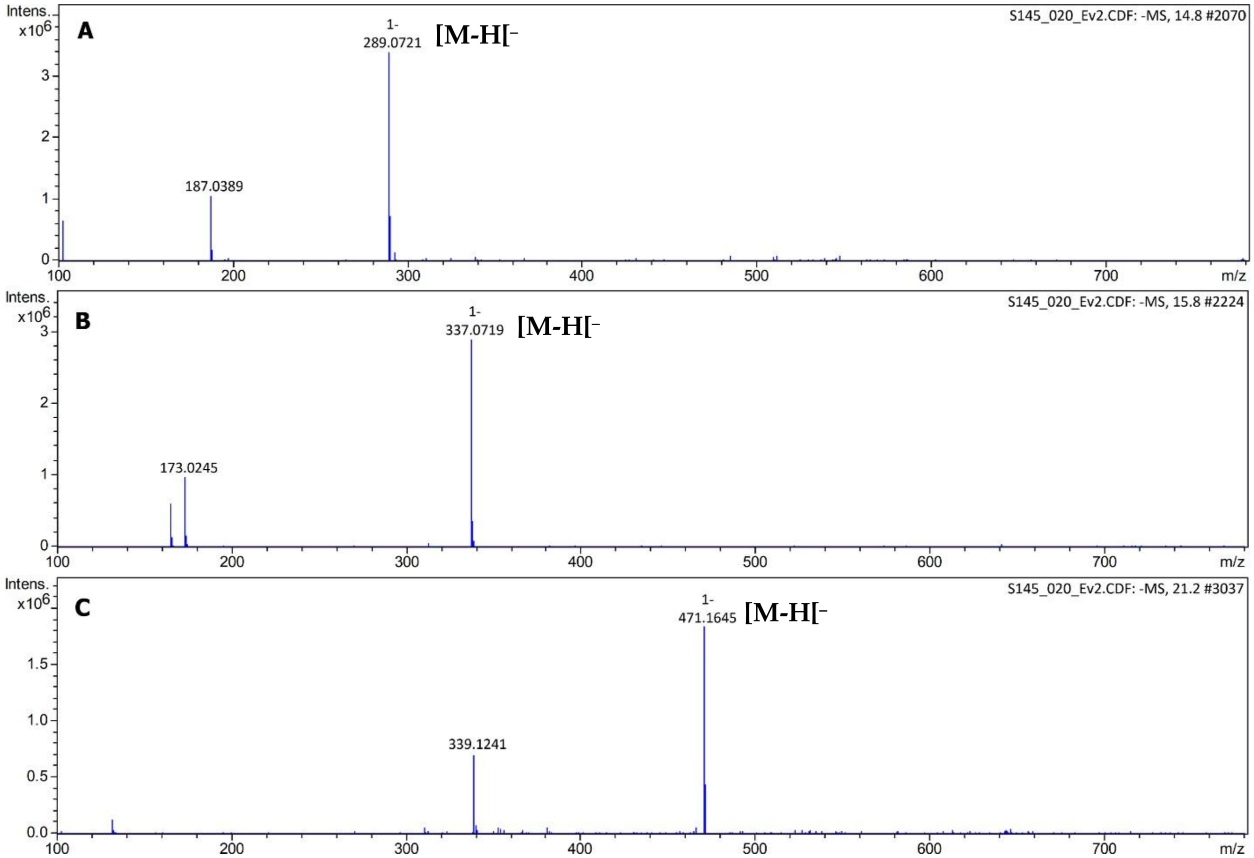

Figure S4. High-resolution mass spectra (HRMS) of the main features for the total ion chromatogram of the S145-derived pigment-rich fraction. A) Compound **1**; B) Compound **2**; C) Compound **3**.
